# Supplementary material for: Survival in macrophages induces enhanced virulence in Cryptococcus
Source: mSphere. 2023 Dec 11;9(1):e00504-23. doi: 10.1128/msphere.00504-23 (PMC10826345; doi:10.1128/msphere.00504-23)
Supplement: Supplemental Materials — Figures S1 and S2 Video S1 legend. [file msphere.00504-23-s0001.pdf]

<u>SUPPLEMENTARY MATERIALS</u>

SUPPELENTARY FIGURES

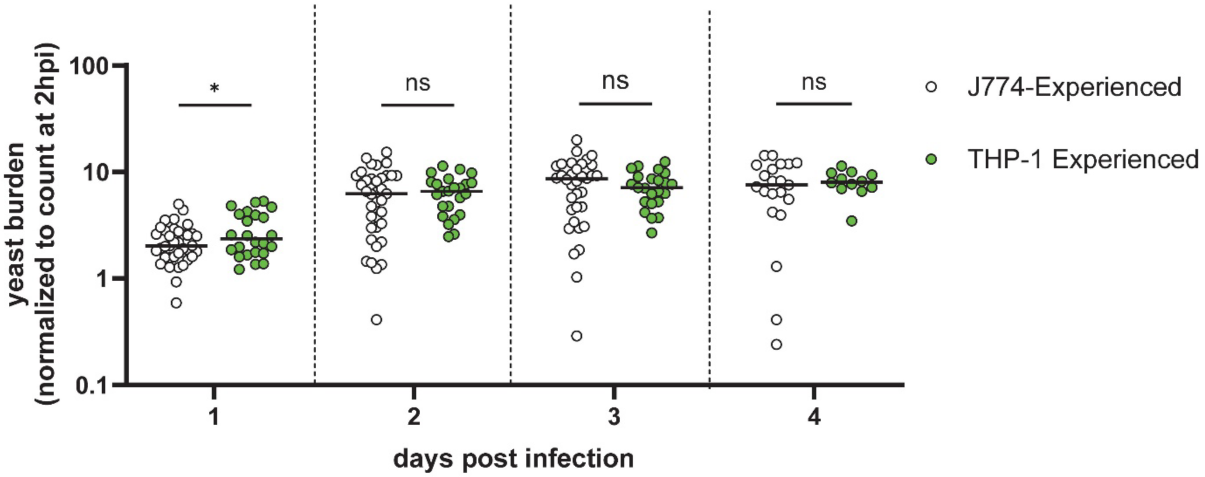

Figure S1. Enhanced virulence in fish is seen in MECs from both murine and human cell lines.
Larvae inoculated with 20-70 fluorescent yeast each as in Fig 1A. Counts were performed via live microscopy at 2hpi and 1 through 4 days post infection (dpi). Daily counts graphed are normalized to count at 2hpi. J774-experienced data is the same as in Figure 1A, compared to THP-1 data separately for clarity. Charted are daily yeast counts normalized to counts at 2hpi. Statistical comparisons represent results of Mann-Whitney tests of log transformed ratios.

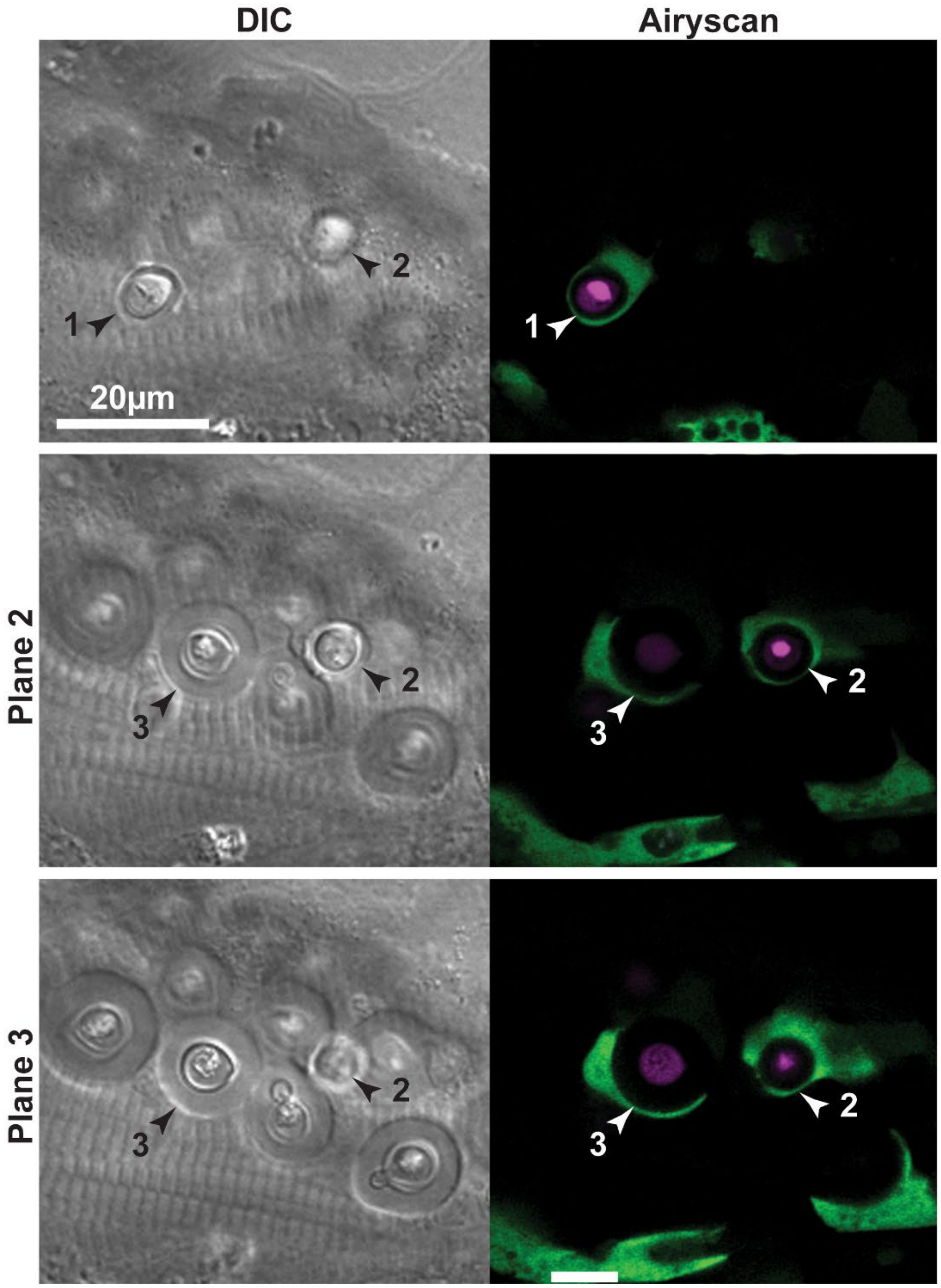

Figure S2. Single slice images of MEC phagocytosis with correlating DIC images.
Same image set as 3D rendering in Figure 2D, with addition of matching DIC images taken within minutes of confocal ones. Individual yeast are numbered to match Figure 2D. Animation of this dataset in Movie S1.

SUPPLEMENTARY MOVIE LEGENDS

Movie S1. Same image depicted in Figures 2D and S2, with rotation to emphasize full internalization of 2 of 3 yeast cells indicated in those figures.
